# Spatial Distribution Patterns in the Very Rare and Species-Rich *Picea chihuahuana *Tree Community (Mexico)

**DOI:** 10.1371/journal.pone.0140442

**Published:** 2015-10-23

**Authors:** Christian Wehenkel, João Marcelo Brazão-Protázio, Artemio Carrillo-Parra, José Hugo Martínez-Guerrero, Felipe Crecente-Campo

**Affiliations:** 1 Instituto de Silvicultura e Industria de la Madera, Universidad Juárez del Estado de Durango, Durango, Mexico; 2 Instituto de Ciências Exatas e Naturais, Faculdade de Estatística, Universidade Federal do Pará, Pará, Brasil; 3 Facultad de Medicina Veterinaria y Zootecnia, Cuerpo Académico de Fauna Silvestre, Universidad Juárez del Estado de Durango, Durango, México; 4 Cerna Ingeniería y Asesoría Medioambiental, S.L.P., Lugo, Spain; Technical University in Zvolen, SLOVAKIA

## Abstract

The very rare Mexican *Picea chihuahuana* tree community covers an area of no more than 300 ha in the Sierra Madre Occidental. This special tree community has been the subject of several studies aimed at learning more about the genetic structure and ecology of the species and the potential effects of climate change. The spatial distribution of trees is a result of many ecological processes and can affect the degree of competition between neighbouring trees, tree density, variability in size and distribution, regeneration, survival, growth, mortality, crown formation and the biological diversity within forest communities. Numerous scale-dependent measures have been established in order to describe spatial forest structure. The overall aim of most of these studies has been to obtain data to help design preservation and conservation strategies. In this study, we examined the spatial distribution pattern of trees in the *P*. *chihuahuana* tree community in 12 localities, in relation to i) tree stand density, ii) diameter distribution (vertical structure), iii) tree species diversity, iv) geographical latitude and v) tree dominance at a fine scale (in 0.25 ha plots), with the aim of obtaining a better understanding of the complex ecosystem processes and biological diversity. Because of the strongly mixed nature of this tree community, which often produces low population densities of each tree species and random tree fall gaps caused by tree death, we expect aggregated patterns in individual *Picea chihuahuana* trees and in the *P*. *chihuahuana* tree community, repulsive *Picea* patterns to other tree species and repulsive patterns of young to adult trees. Each location was represented by one plot of 50 x 50 m (0.25 ha) established in the centre of the tree community. The findings demonstrate that the hypothesis of aggregated tree pattern is not applicable to the mean pattern measured by Clark-Evans index, Uniform Angle index and Mean Directional index of the uneven-aged *P*. *chihuahuana* trees and *P*. *chihuahuana* tree community and but to specific spatial scales measured by the univariate *L*-function. The spatial distribution pattern of *P*. *chihuahuana* trees was found to be independent of patches of other tree species measured by the bivariate *L*-function. The spatial distribution was not significantly related to tree density, diameter distribution or tree species diversity. The index of Clark and Evans decreased significantly from the southern to northern plots containing all tree species. Self-thinning due to intra and inter-specific competition-induced mortality is probably the main cause of the decrease in aggregation intensity during the course of population development in this tree community. We recommend the use of larger sampling plots (> 0.25 ha) in uneven-aged and species-rich forest ecosystems to detect less obvious, but important, relationships between spatial tree pattern and functioning and diversity in these forests.

## Introduction

The endemic *Picea chihuahuana* Martínez, a relict stranded by a warming climate during the current interglacial period [1], is listed as “Endangered” on the “Red List” of threatened species in Mexico [2]. At elevations of between 2,100 and 3,000 m a.s.l., 40 populations comprising at least 42,600 individuals have been detected in three separate clusters in the Sierra Madre Occidental. The size of the populations ranges between 21 and 5,546 individuals, including trees, saplings and seedlings. Individual trees can reach up to 50 m in height and 120 cm in diameter and an age of at least 272 years [3] [1]. The lower branches are almost horizontal, starting at 2 to 5 m in height, while higher branches are extended and somewhat raised, forming a conical crown [4]. *Picea chihuahuana* preferentially inhabits areas of rough terrain located on hillsides and canyons in areas facing northwest or northeast, with slopes ranging from 35% to 80%, at the margins of streams and rivers [5][6]. The species is often associated with other species of the genera *Pinus*, *Quercus*, *Abies*, *Pseudotsuga*, *Populus*, *Prunus*, *Juniperus* and *Cupressus* [6], [7]. The dominant type of disturbance seems to be tree fall gaps in the canopy caused by windstorms, fungi pathogens and insects [1], [8], but not by fire [9].

This very rare pine-spruce-cedar community (hereafter referred to as the *P*. *chihuahuana* tree community) covers an area no more than 300 ha. It remains largely untouched by humans because of its isolated location in very rugged mountainous areas [1], [1], [10]. The *P*. *chihuahuana* tree community has been the subject of several studies aimed at learning more about the genetic structure [3], [7], [10], [11], [12], [13], [14] and ecology of the species [1], [6], [15] and about potential effects of climate change [16], [17], [18]. The overall aim of most of these studies has been to obtain data to help design preservation and conservation strategies [7]. However, the structure (specifically the spatial tree pattern) of the Mexican *Picea chihuahuana* tree community has not yet been analyzed.

Forest structure is both a product and factor of ecosystem processes and biological diversity. Understanding forest structure can therefore help in understanding the history, function and future of a forest ecosystem [19]. Moreover, information about forest structure provides an essential basis for the analysis of forest ecosystem disturbance [20].

Forest structure refers to the patterns and relationships between attributes, including structural type, size, shape and spatial distribution (vertical and horizontal) and also the characteristics of components such as tree crown, foliage, tree bark, tree bole, wood tissue, standing dead trees, fallen trees, roots, pit and mound topography, landscape structure, soil structure, shrub, herb and moss layers, and forest floor and organic layers. Many of these components are fundamental to the functioning and diversity of ecosystems. For example, forest canopies, which differ both vertically and horizontally, are important for intercepting radiation, controlling microclimate and determining habitats [19], [21].

The spatial distribution of trees is a result of many ecological processes and can, for example, affect the degree of competition between neighbouring trees [22], [23], [24], [25], tree density [26], the size distribution and variability, regeneration, survival, growth, mortality and crown formation of forest trees [22], [27], [28], [29] as well as the biological diversity within a forest community [30].

Numerous scale-dependent measures have been established to describe spatial tree structure, such as the Clark-Evans index [31]), Diggle’s *F* and *G*-functions [32], Ripley’s *K*-function [33], [34], [35], the uniform angle index [36], [37], [38] and the mean directional index [39], [40].

In this study, we examined the spatial distribution pattern of trees in the *P*. *chihuahuana* tree community in 12 localities, in relation to i) tree stand density, ii) diameter distribution (vertical structure), iii) tree species diversity, iv) geographical latitude and v) tree dominance at a fine scale (in 0.25 ha plots), with the aim of obtaining a better understanding of the complex ecosystem processes and biological diversity [21]. Because of the strongly mixed nature of this tree community, which often produces low population densities of each tree species and random tree fall gaps caused by tree death, we expect aggregated patterns in individual *P*. *chihuahuana* trees and in the *P*. *chihuahuana* tree community [26], repulsive *Picea* patterns to other tree species and repulsive patterns of young to adult trees [41]. We also assumed no differences between i) the spatial distribution of the northern and southern populations of the *P*. *chihuahuana* tree community, and ii) the spatial distribution of suppressed and dominant trees, because of similar degrees of competition-induced mortality [42].

## Material and Methods

**Fig 1:**
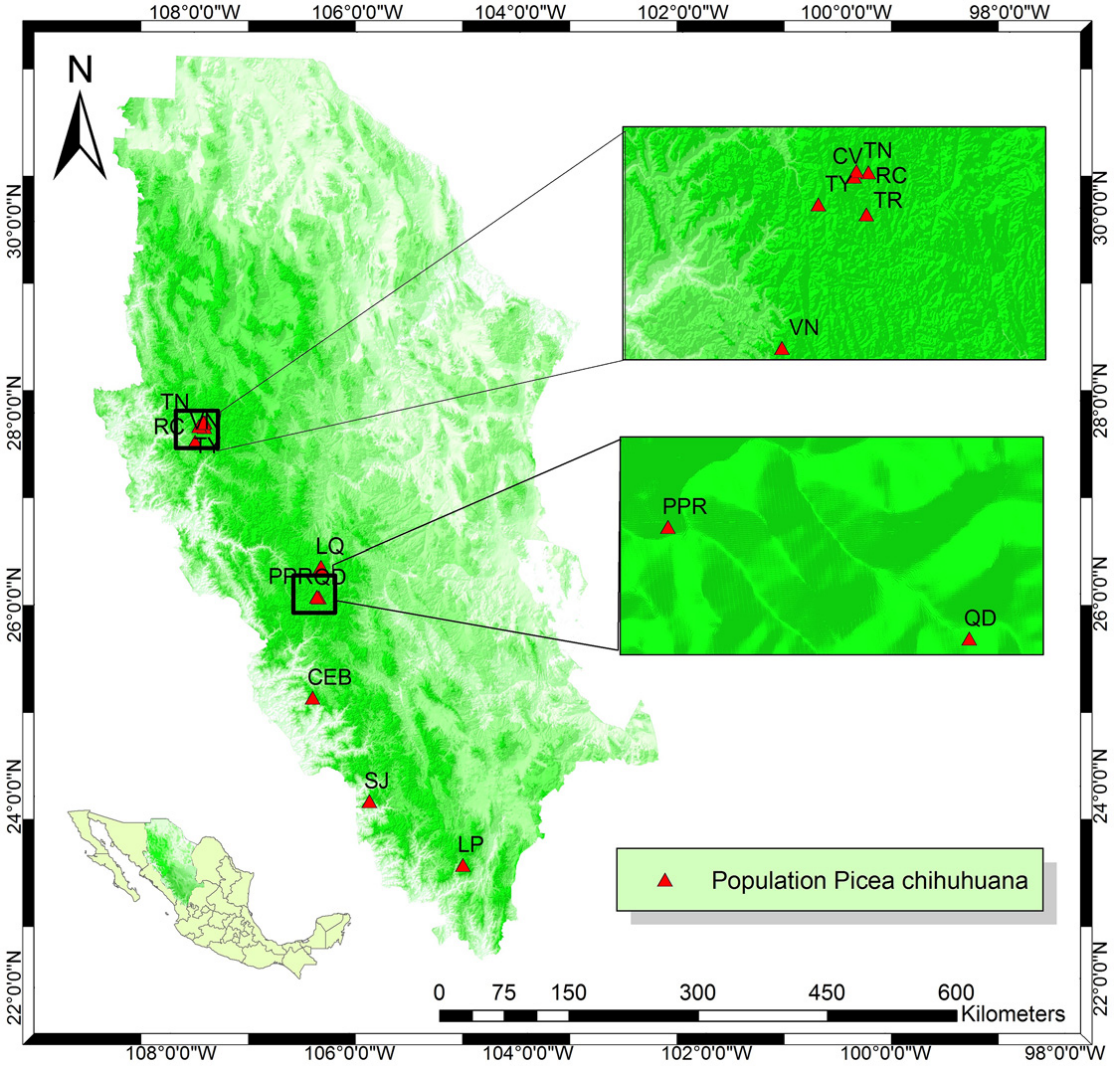
Location of the study area on the Sierra Madre Occidental, Durango (Mexico). Map of the 12 locations of the *Picea chihuahuana* Martínez tree community under study in the States of Durango and Chihuahua (Mexico)**:** 1) La Tinaja (*TN*), 2) El Ranchito (*RC*), 3) El Cuervo (*CV*), 4) Talayote (*TY*), 5) Las Trojas (*TR*), 6) El Venado (*VN*), 7) La Quebrada (*LQ*), 8) Paraje Piedra Rayada (*PPR*), 9) Quebrada de los Durán (Arroyo del Indio Ignacio) (*QD*), 10) Cebollitas (*CB*), 11) San José de las Causas (*SJ*), and 12) La Pista (Arroyo de La Pista) (*LP*).Data sources: Own compilation based on freely-accessible digital maps from INEGI, Mexico (http://www.inegi.org.mx/geo/contenidos/mapadigital/).

We confirm that the field studies provide the specific location of study (Fig 1, S1 Dataset). No vertebrate studies were carried out. Field permit was granted by SEMARNAT, Mexico (http://www.semarnat.gob.mx/).

### Study area

**Fig 2:**
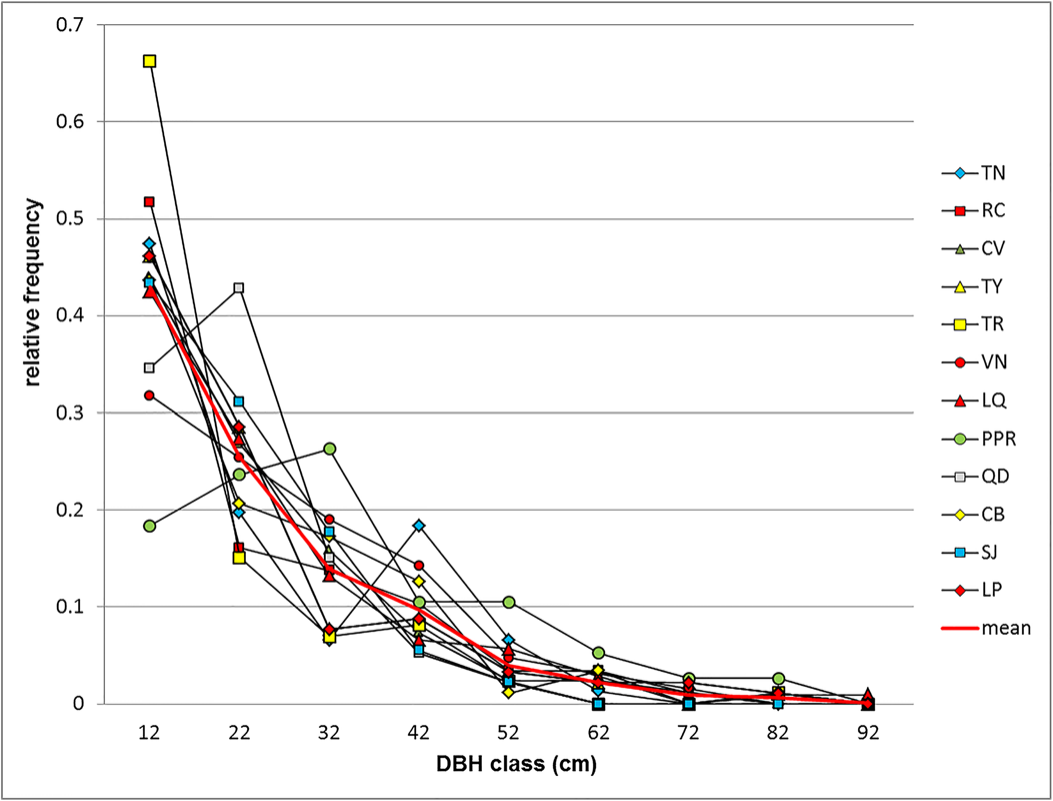
Diameter distribution in the plots representing the 12 locations of the *Picea chihuahuana* Martínez tree community. Diameter distribution in the plots representing the 12 locations of the *Picea chihuahuana* Martínez tree community under study in the States of Durango and Chihuahua (Mexico),considering all tree species.1) La Tinaja (*TN*), 2) El Ranchito (*RC*), 3) El Cuervo (*CV*), 4) Talayote (*TY*), 5) Las Trojas (*TR*), 6) El Venado (*VN*), 7) La Quebrada (*LQ*), 8) Paraje Piedra Rayada (*PPR*), 9) Quebrada de los Durán (Arroyo del Indio Ignacio) (*QD*), 10) Cebollitas (*CB*), 11) San José de las Causas (*SJ*), and 12) La Pista (Arroyo de La Pista) (*LP*).

Chihuahua spruce grows in areas characterized by an average temperature of between 9 and 12°C [1], precipitation ranging from 600 mm to 1,300 mm [43] and soil pH of 5.3–6.3 [4]. In order to determine the spatial tree structure of the *P*. *chihuahuana* tree community, 12 locations where the community occurs in the State of Durango and Chihuahua (north-western Mexico) were considered (Fig 1). Each location was represented by one plot of 50 x 50 m (0.25 ha) established in the centre of the tree community. Trees of all species of diameter at breast height (DBH) ≥ 7 cm were fully scored. The DBH, height and x, y coordinates were also recorded. The stem number per hectare (*N*), stand basal area (*G*), quadratic mean diameter (*dg*), mean breast height diameter (*d*), mean total height (*h*), maximum diameter (*d*_*max*_), and maximum height (*d*_*max*_) of all tree species (total) and *Picea chihuahuana* M. (Pch) were computed (Table 1). The total numbers of tree species within each of the populations in the *P*. *chihuahuana* tree community have been reported by Quiñones-Pérez et al.[44].The DBH structures (as parameters of vertical structure) in the 12 plots considering all tree species showed a reverse *J*-shaped form (Fig 2) typical of uneven-aged forests. Fig 2 also demonstrates that the minimum balanced structure area of this tree community is very small (< 3ha) [45]. In total, 15 tree species were foundin the 12 plots: *Abies durangensis* Martínez, *Cupressus lindleyi* Klotzsch ex Endl., *Juniperus deppeana Steud*., *P*. *chihuahuana*, *Pinus arizonica* Engelm., *Pinus strobiformis* Engelm., *Pinus cooperi* Martínez, *Pinus durangensis Martínez*, *Pinus leiophylla* Schl. & Cham., *Pinus teocote* Schiede ex Schltdl. & Cham., *Populus tremuloides* Michx., *Prunus serotina* Ehrh., *Pseudotsuga menziesii* (Mirb.) Franco, *Quercus sideroxyla* Humb.and*Quercus crassifolia* Humb. In each plot, *P*. *chihuahuana* grew along with three to eight other tree species [44]. To represent the diversity profile (*v*_*sp*,*a*_) of the tree species, we selected the described diversity for each location. Thus, each location of the *P*. *chihuahuana* tree community was characterized by the total number of tree species (species richness, (*υ*_*sp*,*0*_)), effective number of tree species (Simpson index, (*υ*_*sp*,*2*_)) and the number of prevalent tree species (*υ*_*sp*,*∞*_), as Hill numbers [46] in each plot. The diversity values were taken from [13].

**Table 1:** Summary of important stand parameters calculated from the tree data: stem number per hectare (*N*), stand basal area (*G*), quadratic mean diameter (*dg*), mean breast height diameter (*d*), mean total height (*h*), maximum diameter (*dmax*), and maximum height (*dmax*) of the all tree species (total) and *Picea chihuahuana* M. (Pch) in the 50 x 50 m plotsin the 12 study locations and minimum (min), mean and maximum (max) parameter values for the stands. 1) La Tinaja (*TN*), 2) El Ranchito (*RC*), 3) El Cuervo (*CV*), 4) Talayote (*TY*), 5) Las Trojas (*TR*), 6) El Venado (*VN*), 7) La Quebrada (*LQ*), 8) Paraje Piedra Rayada (*PPR*), 9) Quebrada de los Durán (Arroyo del Indio Ignacio) (*QD*), 10) Cebollitas (*CB*), 11) San José de las Causas (*SJ*), and 12) La Pista (Arroyo de La Pista) (*LP*).

	*N*		*G*		*Dg*		*d*		*h*		*d*_*max*_		*h*_*max*_	
	[N/ha]		[m^2/^ha]		[cm]		[cm]		[m]		[cm]		[m]	
Code	total	Pch	total	Pch	Total	Pch	total	Pch	total	Pch	total	Pch	total	Pch
TN	304	132	19.0	14.3	28.2	37.2	24.3	34.3	13.0	18.9	59.0	59.0	31.7	31.7
RC	348	44	20.2	5.6	27.2	40.1	22.2	35.5	12.5	21.1	74.8	63.4	32.3	32.3
CV	328	88	18.1	6.2	26.5	30.0	22.5	26.0	12.5	15.7	68.8	66.2	32.3	32.3
TY	232	112	18.5	10.6	31.9	34.8	28.1	31.4	17.3	19.9	60.0	60.0	46.0	46.0
TR	356	48	13.7	2.5	22.1	25.8	17.9	23.6	9.7	14.0	80.0	43.0	24.1	24.1
VN	260	108	18.1	11.5	29.8	36.9	25.9	32.4	13.7	18.8	67.0	67.0	38.2	38.2
LQ	432	140	27.8	13.3	28.1	34.8	23.9	31.0	14.5	18.3	93.8	77.6	33.4	33.4
PPR	152	92	16.6	8.2	37.3	33.6	32.9	29.8	17.9	16.6	79.0	57.5	36.5	36.5
QD	532	92	23.3	5.6	23.6	27.9	21.6	25.2	14.1	16.6	55.0	55.0	31.5	31.5
CB	352	44	21.8	2.1	28.1	24.7	23.8	20.8	14.6	12.0	82.0	41.8	31.2	24.8
SJ	360	48	15.2	1.7	23.2	21.0	20.8	19.5	13.8	14.2	55.2	34.0	36.9	24.7
LP	364	96	22.9	10.7	28.3	37.7	23.4	29.6	14.9	15.9	78.0	78.0	40.0	40.0
**min**	**152**	**44**	**13.7**	**1.7**	**22.1**	**21.0**	**17.9**	**19.5**	**9.7**	**12.0**	**55.0**	**34.0**	**24.1**	**24.1**
**mean**	**335**	**87**	**19.6**	**7.7**	**27.9**	**32.0**	**23.9**	**28.3**	**14.0**	**16.8**	**71.1**	**58.5**	**34.5**	**33.0**
**max**	**532**	**140**	**27.8**	**14.3**	**37.3**	**40.1**	**32.9**	**35.5**	**17.9**	**21.1**	**93.8**	**78.0**	**46.0**	**46.0**

### Spatial Structural Analysis

#### Clark-Evans index (CE), Uniform Angle index ($\bar{W}$) and Mean Directional index ($\bar{R}$)

The Clark-Evans index (*CE*) [31], the Uniform Angle index ($\bar{W}$ **)** [36] and the Mean Directional index ($\bar{R}$) [47] were used to describe the spatial distribution of the trees in each study plot, on the basis of the spatial distribution of the *n* trees nearest to a reference tree *i*. The *CE* was estimated using one neighbour (*n* = 1), while $\bar{W}$ and $\bar{R}$ were calculated using four neighbours (*n* = 4) [48], [49], [40]. A Poisson distribution pattern was characterised by a *CE* value of 1, cluster tendency by *CE* < 1 and a tendency of regular distribution of trees by *CE* > 1, with a maximum of 2.1491 for a hexagonal arrangement of trees.

For calculation of $\bar{W}$, *W*_*i*_ must first be calculated. The angle *α*_0_ was set at 72 degrees, which yielded a mean value of $\bar{W}$ = 0.5 [37]. For each tree, the value of *W*_*i*_ was determined and the average $\bar{W}$ for all trees was calculated. *W*_*i*_ and $\bar{W}$ values close to 0 were associated with a regular neighbourhood of tree *i*, while values of *W*_*i*_ and $\bar{W}$ close to 1 corresponded to irregularity of the spatial distribution in the neighbourhood of tree *i*.

Finally, calculation of $\bar{R}$ also requires calculation of *R*_*i*_. The exact value of $\bar{R}$ for a Poisson distribution in each plot in a 4-tree sample was 1.799, as obtained by a simulation based on 10^6^ trees. Values of *R*_*i*_ and plot mean $\bar{R}$ close to 0 were associated with a regular tendency of the neighbourhood of tree *i*, while values of *R*_*i*_ and $\bar{R}$ larger than 1.799 were associated with a tendency of the spatial distribution in the neighbourhood of tree *i* to be irregular (see more in [40]).

To exclude the edge effect, and therefore to enhance the accuracy of the estimates, the nearest-neighbour edge-correction concept (NN1) was applied, as proposed by [29], for calculating *CE*, $\bar{W}$ and $\bar{R}$.

The hypothesis of complete spatial randomness (CSR) for the mean values of *CE*, $\bar{W}$ and $\bar{R}$ for each plot was tested by atwo-sided permutation test (here 10,000 permutations) If 1—*P*(*Z* ≥ *CE*), *P*(*Z* ≥ $\bar{W}$), and *P*(*Z* ≥ $\bar{R}$) are non-significantly small or non-significantly high (i.e. 0.01 < *P* < 0.99, at the 1% acceptance level), we can expect random effects and otherwise, directed forces. If the observed 1—*P*(*Z* ≥ *CE*), *P*(*Z* ≥ $\bar{W}$) or *P*(*Z* ≥ $\bar{R}$) values are smaller than 0.01, we can assume non-randomly acting diversifying forces (e.g. seed dispersal pattern, association with nutrient-rich patches) that will produce a clustered distribution. If the observed 1—*P*(*Z* ≥ *CE*), *P*(*Z* ≥ $\bar{W}$) or *P*(*Z* ≥$\bar{R}$) values are larger than 0.99, we assume that non-randomly acting homogenizing forces (e.g. competition for light, water and nutrients) will yield a regular distribution [50], [51],[40]. After Bonferroni correction[52], the new (modified) critical *P* value (significance level* = 0.0002) was calculated by dividing the critical *P* value (here the significance level = 0.05) by the number of comparisons (hypotheses) (*m* = 216).

#### Spatial Structural Analysis by Ripley’s K(t)-function

Ripley’s *K(t)-*function is used to characterize completely mapped spatial point process data. The mapped data are usually recorded in two or three dimensions and include the locations of all events in a predefined study area. Unlike other functions (e.g. mean nearest-neighbour distance or the cumulative distribution function of distance from random points to their nearest neighbours), this function preserves information about distances between all points in the pattern, thus enabling visualization of how point pattern distributions vary with scale. Ripley’s *K(t)*function is useful for summarizing point patterns, testing hypotheses about the patterns, estimating parameters and fitting models. Bivariate or multivariate *K(t)* functions can be used to describe relationships between two or more point patterns [53].

Ripley’s *K-*function [33], [35] was used to determine the scales at which the tree pattern in each plot tends to be regular, clumped or random. The function was used to describe the relationship between the spatial pattern of *Picea chihuahuana* and the spatial structure of the other tree species inside the 12 plots.

The univariate Ripley's *K*-function [53] can be estimated as

$$K_{U}\left(r\right)=\frac{A}{n^{2}}\sum \;\sum w_{ij}\;\left(r\right)\delta \left(d_{ij}<r\right),$$ (1)

where *A* is the area of the study region, *n* is the number of observed points, *w*_*ij*_(*r*) is an edge effect correction factor, *δ(r)* is an indicator function and *d*_*ij*_ is the distance between the *i*-th and *j*-th points.

Because of its hyperbolic behavior, the interpretation of the *K*-function is not straightforward and a modification, called the *L*-function, was proposed by Besag (1977) in order to normalize the function (Besag, 1977):

$$L_{U}\left(r\right)=\sqrt{\frac{K_{U}\left(r\right)}{\pi }}-r.$$ (2)

Now, the expected value of the univariate *L*-function under CSR is 0 for all *r*, positive when the pattern tends to be clustered and negative when the pattern tends to be regular.

In order to test the deviation from randomness of the point pattern using the univariate *L*-function, a 99% simulation envelope of *L*(*r*) was computed, using the Monte Carlo Method [54], from 1,000 simulated CSR patterns with the same number of points contained inside a region with the same geometry.

The bivariate Ripley’s *K*-function [53] is estimated as

$$K_{ij}\;=\;\frac{A}{n_{i}n_{j}}\sum \;\sum \;w_{ik,jl}\left(r\right)\delta \left(d_{ik,jl}<r\right),$$ (3)

where n_*i*_ and n_*j*_ are the numbers of the points of type *i* and *j* respectively, *w*_*ik*,*jl*_(*r*) is an edge effect correction factor and *δ(r)* is an indicator function and *d*_*ik*,*jl*_ is the distance between the *k*-th point of type *i* and the *l*-th point of type *j*.

Due to the edge effect, *K*_*ij*_ and *K*_*ji*_ are correlated, but not identical, and therefore the following means of estimating *K*_*B*_ is recommended:

$$K_{B}\left(r\right)=\frac{n_{i}K_{ij}\left(r\right)+n_{j}K_{ji}\left(r\right)}{n_{i}+n_{j}}.$$ (4)

Its associated bivariate *L*-function is defined as

$$L_{B}\left(r\right)=\sqrt{\frac{K_{B}\left(r\right)}{\pi }}-r.$$ (5)

The expected value of the bivariate *L*-function under spatial independence is 0 for all *r*, positive when the two point processes tend to be aggregated and negative when the two point processes tend to be repulsive.

In order to generate the simulation envelope that corresponds to the hypothesis of spatial independence, the method holds the point pattern of type 1 and type 2 constant and then randomizes their relative position in each simulation. For more details, see Lotwick and Silverman [55].

The trees were grouped in two diameter classes to check i) whether clustering at small scales was caused by a high degree of aggregation of smaller trees (using the univariate *L*-function) and ii) whether young trees were clustered around the adults (using the bivariate *L*-function). To find the *d*_*cut*_ (cut-off) that divides the population into smaller and larger individuals (determined by their DBH), we obtained the bivariate *L*-function for various values within the range 7 < *d*_*cut*,*DBH*_ < 40 cm and we therefore chose the *d*_*cut*_ where the aggregation patterns between the groups were visually more significant.

*d*_*cut*,*DBH*,*all*_ corresponded to 23.2 cm, *d*_*cut*,*DBH*,*Pch*_ to 29.3 cm.

All analyses were performed using the "Spatstat" package implemented in the free statistical application *R*[56][57].

The statistical tests for spatial tree pattern, null hypothesis, interpretation and related ecological questions are summarised in Table 2.

**Table 2:** Summary of statistical tests of spatial tree structure, null hypothesis, interpretation and related ecological questions; *CE* = neighbourhood-based Clark-Evans index, $\bar{W}$ = Uniform Angle index, $\bar{R}$ = Mean Directional index, LU(r) = univariate L-function, and *LB*(*r*) = bivariate *L*-function.

*CE*	$\bar{W}$	$\bar{R}$	*L*-function	*CE*, $\bar{W},\;\bar{R}$, *L*_*U*_(*r*)	*L*_*B*_(*r*)
*CE* = 1	$\bar{W}$ = 0.5	$\bar{R}$ = 1.799	*L*(*r*) = 0	Complete Spatial Randomness	Independence
*CE*< 1	$\bar{W}$> 0.5	$\bar{R}$> 1.799	*L*(*r*) > 0	Clustering	Aggregation
*CE*> 1	$\bar{W}$< 0.5	$\bar{R}$< 1.799	*L*(*r*) < 0	Regularity	Repulsion
Null hypotheses				Aggregation of (young and larger) trees	Repulsive patterns of young tree to adults/Repulsive *Picea chihuahuana* patterns to other tree species
Alternative hypothesis				Absence of spatial relationship between individuals	Independence between species or between cohorts interaction

### Covariation analysis

The relationships between stand densities (N and G), relative frequency of 10 cm DBH class (fcd) and tree species diversities (the Hill numbers υsp,0, υsp,2, andυsp,∞ [46]), degree of latitude (lat) and spatial pattern indices (CE, $\bar{W}$ and $\bar{R}$) were measured by the covariation (C) described by Gregorius et al. [50]. This method can detect types of covariation that are monotonous but not necessarily linear. C ranges between -1 and 1, where C = 1 indicates an entirely positive covariation and C = -1 a strictly negative covariation. If the denominator is zero, C is undefined [50]. Formally,

$$C\;=\frac{\sum_{i<j}\left(X_{i}-X_{j}\right)\cdot (Y_{i}-Y_{j})}{\sum_{i<j}\left|\left(X_{i}-X_{j}\right)\cdot (Y_{i}-Y_{j})\right|}.\;\text{C}≔\text{i}<\text{j}(\text{Xi-Xj})\cdot (\text{Yi-Yj})\text{i}<\text{j}(\text{Xi-Xj})\cdot (\text{Yi-Yj})$$ (6)

In order to test the possibility that the observed degrees of covariation *C[N x CE]*, *C[N x* $\bar{W}$ *]*, *C[N x* $\bar{R}$ *], C[G x CE]*, *C[G x* $\bar{W}$ *]*, *C[G x* $\bar{R}$ *], C[fcd x CE]*, *C[fcd x* $\bar{W}$ *]*, *C[fcd x* $\bar{R}$ *], C[υ*_*sp*_ *x CE]*, *C[υ*_*sp*_ *x* $\bar{W}$ *]*, *C[υ*_*sp*_ *x* $\bar{R}$ *]*, *C[lat x CE]*, *C[lat x* $\bar{W}$ *]* and *C[lat x* $\bar{R}$ *]* were only produced by random events rather than directed forces, a one-sided permutation test was performed (here 10,000 permutations) [58]. After Bonferroni correction, the new critical *P* value was 0.002.

In order to test whether the observed differences in the average spatial pattern indices (*Diff*) (*CE*, $\bar{W}$ and $\bar{R}$) between i) *P*. *chihuahuana* trees and all other tree species, ii) suppressed and dominant *P*. *chihuahuana* trees and iii) suppressed and dominant trees of all species in the plots were produced solely by random events rather than directed forces, a permutation test based on 10,000 randomly chosen reassignments was performed [58]. Loosely based on the *BAL* competition Index [59] [60], the dominant tree class used in the present study included all larger trees that together included 50% of the stand basal area. The suppressed trees included all smaller trees that together included the other 50% of the stand basal area. This permutation test constitutes a non-parametric approach, which among other uses enables comparison of two groups in terms of the mean values of some variable; however, unlike with the *t* test, the assumptions of normality and equality of variances do not need to be satisfied by the data [51]. After Bonferroni correction of the data, the new critical *P* value was 0.007.

## Results

Most of the *P*. *chihuahuana* trees were randomly distributed (92% of cases), as confirmed by all measures used (and considering a significance level of 1%): the Clark-Evans index (*CE*), uniform angle index ($\bar{W}$), mean directional index ($\bar{R}$) and univariate *L*-function. Based on *CE*, $\bar{W}$ and $\bar{R}$, 8% of the plots display clusteringat the 1% significance level. The univariate *L*-function indicated CSR in all plots, mostly due to the low effective tree number (repetitions) for calculating the values (Table 3). The bivariate *L*-function showed that the number of *Picea* trees smaller than 29.3 cm DBH in the neighborhood of larger *Picea* trees (or equivalently the number of larger *Picea* trees in the neighbourhood of smaller *Picea* trees) was only larger than expected in TN and CV (Table 4). However, after Bonferroni correction of the data, all indices indicated random distribution of the *P*. *chihuahuana* trees in all plots.

**Fig 3:**
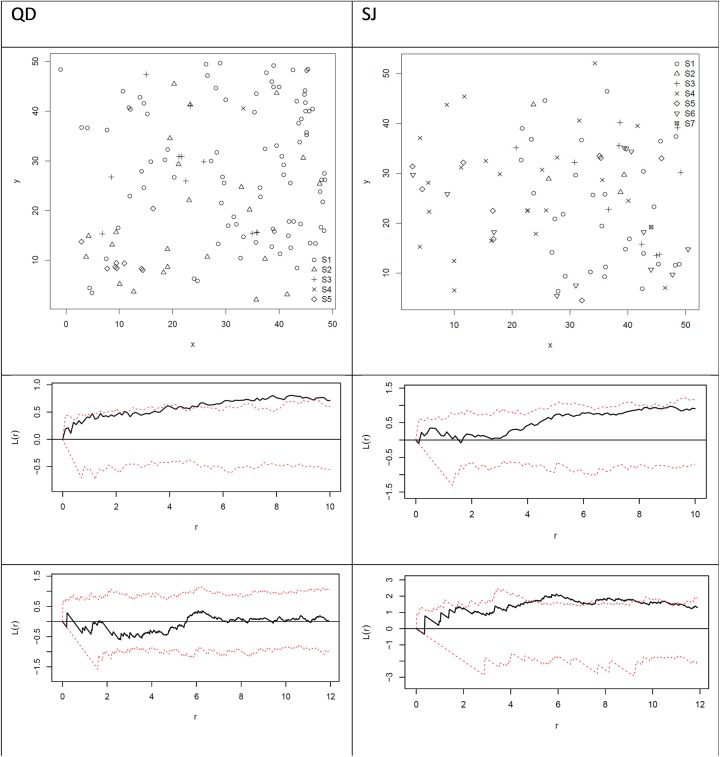
Sample plots (50 x 50 m) illustrating the location of each tree, univariate and bivariate *L*-functions. Sample plots (50 x 50 m) illustrating the location of each tree of diameter at breast height (DBH) above 7 cm in the very rare and species-rich *Picea chihuahuana* tree community in Quebrada de los Durán (QD) and San José de las Causas (SJ), Durango (Mexico). S1 represents the *P*. *chihuahuana* trees (above), and S2–S7 represent the other tree species. For the point patterns in the QDand SJ plots, univariate *L*-functions are represented by black lines. The 99% simulation envelope (dashed red lines) for the CSR hypothesis was calculated via the Monte Carlo Method (Besag 1977), with 1,000 simulations (central). For the point pattern in the QD and SJ plots, the bivariate L-function is indicated represented by black lines. The 99% simulation envelope (dashed red lines) for the Independence hypothesis was calculated by the Random Shifting Method (Lotwick & Silverman, 1983), with 1,000 simulations (below).

Considering all tree species in each plot, the *CE* indicated CSR in 67% of the plots. The $\bar{W}$ and $\bar{R}$ indicate CSR in 92% of the plots at the 1% significance level (Table 5). The univariate *L*-function shows that the trees of all species in 42% of the plots are completely randomly distributed. For all trees and trees smaller than 23.2 cm DBH, a considerable proportion (42%) of the plots demonstrated clustering at smaller scales. For trees equal or larger than 23.2 cm DBH, all plots show CSR. For all trees, a smaller proportion (17%) of the plots indicate tree clustering at the intermediate and larger scales (Table 4, Fig 3 (above and centre)). The bivariate *L*-function demonstrated that the number of smaller trees in the neighbourhood of larger trees (or equivalently the number of larger trees in the neighbourhood of smaller trees) was larger than expected (Table 4). However, after Bonferroni correction, the data indicated that the trees in 92% of the plots were randomly distributed (*P* ≥ 0.0002).

**Table 3:** Spatial structure of *Picea chihuahuana* M. in the 50 x 50 m plots in the 12 study locations, based on the neighbourhood-based Clark-Evans index (*CE*), Uniform Angle index ($\bar{W}$), and Mean Directional index ($\bar{R}$) (*P* values estimated with 10,000 permutations) and univariate *L*-function . The 99% simulation envelope (dashed red lines) for the CSR hypothesis was calculated by the Monte Carlo Method (Besag 1977), with 1,000 simulations (distance interval equals 0–12 m). *N* equals the number of *Picea chihuahuana* M. trees in the plot. 1) La Tinaja (*TN*), 2) El Ranchito (*RC*), 3) El Cuervo (*CV*), 4) Talayote (*TY*), 5) Las Trojas (*TR*), 6) El Venado (*VN*), 7) La Quebrada (*LQ*), 8) Paraje Piedra Rayada (*PPR*), 9) Quebrada de los Durán (Arroyo del Indio Ignacio) (*QD*), 10) Cebollitas (*CB*), 11) San José de las Causas (*SJ*), and 12) La Pista (Arroyo de La Pista) (*LP*).

Location	*N*	*CE*	*1-P* ****(**** *Z* ****≥**** *CE* ****)****		$\bar{W}$	*P*(*Z* ≥ $\bar{W}$)	$\bar{R}$	*P*(*Z* ≥ $\bar{R}$)	Univariate *L*-function
**TN**	33	0.704	0.0525	0.575		0.0704	2.354	0.0245	CSR
**RC**	11	0.469	0.0653	1.000		0.0034	3.651	0.0026	CSR
**CV**	22	0.465	0.0006	0.469		0.6556	1.892	0.3641	CSR
**TY**	28	0.728	0.0504	0.510		0.2850	1.761	0.3946	CSR
**TR**	12	0.566	0.0309	0.488		0.4724	1.816	0.3078	CSR
**VN**	27	0.691	0.0533	0.539		0.1992	1.833	0.3612	CSR
**LQ**	35	0.676	0.0340	0.477		0.7209	1.665	0.7712	CSR
**PPR**	23	0.856	0.7443	0.472		0.4973	1.361	0.7714	CSR
**QD**	23	0.849	0.5424	0.348		0.9888	1.213	0.9513	CSR
**CB**	11	1.152	0.9342	-		-	-	-	CSR
**SJ**	12	0.547	0.0639	-		-	-	-	CSR
**LP**	24	0.744	0.2880	0.516		0.3941	1.972	0.3546	CSR
**mean**	22	0.704	0.2383	0.539		0.4287	1.952	0.4303	

Note: Significant results after Bonferroni correction are shown in bold type. $\bar{W}$ and $\bar{R}$ failed in some plots because of an insufficient number of trees (repetitions) for the calculations.

**Table 4:** Analysis of spatial tree structure in 50 x 50 m plots in the twelve locations including all tree species using the univariate (for all trees, smaller trees of all species [44](< 23.2 cm diameter at the breast height (DBH)), larger trees (≥ 23.2 m DBH), and bivariate versions of the L-function (spatial pattern of *Picea chihuahuana* (Pch) vs. other tree species, pattern of smaller vs. larger trees and of smaller Pch (< 29.3 cm DBH) vs. larger Pchtrees (≥ 29.3 cm DBH)). The 99% simulation envelope (dashed red lines) for the CSR hypothesis (for the univariate *L*-function) and for spatial independence hypothesis (for the bivariate *L*-function) was calculated by the Monte Carlo Method (Besag 1977), with 1,000 simulations (distance interval equals 0–12 m).1) La Tinaja (*TN*), 2) El Ranchito (*RC*), 3) El Cuervo (*CV*), 4) Talayote (*TY*), 5) Las Trojas (*TR*), 6) El Venado (*VN*), 7) La Quebrada (*LQ*), 8) Paraje Piedra Rayada (*PPR*), 9) Quebrada de los Durán (Arroyo del Indio Ignacio) (*QD*), 10) Cebollitas (*CB*), 11) San José de las Causas (*SJ*), and 12) La Pista (Arroyo de La Pista) (*LP*).

****Location****	****Univariate**** *L* ****-function****			****Bivariate**** *L* ****-function****		
	****for all trees****	****smallertrees****	****larger trees****	****Pch vs. other tree species****	****smaller vs. larger tres****	****smaller vs. larger Pch trees****
**TN**	Clustering at *r* = 0.75 m	Clustering at *r* = 1m	CSR	Independence	Independence	*r* = 4 m
**RC**	Clustering at *r* < 1.75 m	Clustering at 0 < *r* < 2 m	CSR	Independence	Aggregation for 0 < *r* <2 m	Independence
**CV**	Clustering at 2 < *r* < 8 m	Clustering at 2< *r* < 6 m and *r* > 13 m	CSR	Independence	Aggregation for *r* > 2 m	*r* = 1 m
**TY**	CSR	CSR	CSR	Independence	Aggregation for 0 < *r* < 2 m	Independence
**TR**	Clustering at *r* = 3 m	Clustering at *r* < 1 m	CSR	Independence	Aggregation in 0 < *r* < 1 and 2.5 < *r* < 4 m	Independence
**VN**	Clustering at *r* = 0.75 m	Clustering at *r* = 1 m	CSR	Independence	Aggregation for0 < *r* < 1 m	Independence
**LQ**	Clustering at *r* > 4 m	Clustering at *r* < 1 m	CSR	Independence	Aggregation for *r* > 2 m	Independence
**PPR**	CSR	-	-	Independence	Independence	Independence
**QD**	Clustering at *r* > 6 m	CSR	CSR	Independence	Aggregation for *r* > 1 m	Independence
**CB**	CSR	CSR	CSR	Independence	Aggregation for 0 < *r*< 1 m	Independence
**SJ**	CSR	Clustering at *r* = 1 m	CSR	Aggregation at *r* = 5 m	Independence	Independence
**LP**	CSR	CSR	CSR	Independence	Aggregation for *r* = 1 m	Independence

**Table 5:** Analysis of spatial tree structure in 50 x 50 m plots in the 12 locations including all tree species (species shown [44]) and based on the neighbourhood-based Clark-Evans index (*CE*), Uniform Angle index ($\bar{W}$), and Mean Directional index ($\bar{R}$). *P* values estimated with 10,000 permutations. 1) La Tinaja (*TN*), 2) El Ranchito (*RC*), 3) El Cuervo (*CV*), 4) Talayote (*TY*), 5) Las Trojas (*TR*), 6) El Venado (*VN*), 7) La Quebrada (*LQ*), 8) Paraje Piedra Rayada (*PPR*), 9) Quebrada de los Durán (Arroyo del Indio Ignacio) (*QD*), 10) Cebollitas (*CB*), 11) San José de las Causas (*SJ*), and 12) La Pista (Arroyo de La Pista) (*LP*).

Location	*N*	Species number	*CE*	*1-P*(*Z* ≥ *CE*)	$\bar{W}$	*P*(*Z* ≥ $\bar{W}$)	$\bar{R}$	*P*(*Z* ≥ $\bar{R}$)
**TN**	76	7	0.711	0.0011	0.503	0.3060	2.006	0.0697
**RC**	87	7	0.744	0.0017	0.554	0.0186	2.072	0.0212
**CV**	82	5	0.745	0.0042	0.598	0.0012	**2.34** ^**cl**^	**0.0002**
**TY**	58	7	0.786	0.0412	0.524	0.1832	1.942	0.2009
**TR**	89	9	0.748	0.0063	0.536	0.1165	2.010	0.1171
**VN**	65	8	0.857	0.1890	0.538	0.1478	1.786	0.6080
**LQ**	112	7	0.852	0.1988	0.519	0.1991	1.952	0.1219
**PPR**	38	4	0.897	0.8792	0.455	0.8316	1.658	0.7051
**QD**	133	5	0.800	0.0164	0.549	0.0154	2.042	0.0275
**CB**	88	8	0.848	0.1762	0.552	0.0441	2.119	0.0291
**SJ**	90	7	0.890	0.3701	0.485	0.5558	1.809	0.3687
**LP**	91	5	0.902	0.3781	0.481	0.7661	1.789	0.6002
**mean**	84	6.6	0.704	0.2383	0.539	0.4287	1.952	0.4303

Note: Significant results after Bonferroni correction are shown in bold type. ^cl^ indicates a clustering pattern in the plot.

The *Picea* and other tree species were not spatially segregated, i.e. *Picea* tended to be found in patches of other tree species excepting the location San Jose de las Causas (SJ) (Table 4, Fig 3 at the bottom left and right).

Analysis of the suppressed or immature trees versus the dominant or mature trees revealed that at the 1% significance level and according to *CE*, suppressed *P*. *chihuahuana* M. trees occurred in three plots (25%) and suppressed trees of all species occurred in five plots (41.7%), thus demonstrating clustering. However, dominant trees showed CSR in all 12 plots (Tables 6 and 7). The $\bar{W}$ and $\bar{R}$ values indicated CSR for all the plots where they were obtained. However, the mean $\bar{W}$ and $\bar{R}$ values for all plots of the suppressed trees (of all species) were statistically significantly higher than the dominant trees (of all species) (*P* = 0.0009 and *P* = 0.0006, respectively), also after Bonferroni correction (*P* < 0.007). Therefore, the tendency for clustering was significantly higher in the suppressed trees than in the dominant trees. $\bar{W}$ and $\bar{R}$ failed in some plots because of an insufficient number of trees(repetitions) for the calculations.

**Table 6:** Analysis of spatial structure of the suppressed and dominant trees in 50 x 50 m plots containing all *Picea chihuahuana M*. trees, in the 12 study locations, based on the neighbourhood-based Clark-Evans index (*CE*), Uniform Angle index ($\bar{W}$), and Mean Directional index ($\bar{R}$). *P* values estimated with 10,000 permutations. *N* equals the tree number in the plot. 1) La Tinaja (*TN*), 2) El Ranchito (*RC*), 3) El Cuervo (*CV*), 4) Talayote (*TY*), 5) Las Trojas (*TR*), 6) El Venado (*VN*), 7) La Quebrada (*LQ*), 8) Paraje Piedra Rayada (*PPR*), 9) Quebrada de los Durán (Arroyo del Indio Ignacio) (*QD*), 10) Cebollitas (*CB*), 11) San José de las Causas (*SJ*), and 12) La Pista (Arroyo de La Pista) (*LP*).

Location	*N*	*CE*	*1-P*(*Z* ≥ *CE*)	$\bar{W}$	*P*(*Z* ≥ $\bar{W}$)	$\bar{R}$	*P*(*Z* ≥ $\bar{R}$)
Suppressed *Picea chihuahuana* M. trees							
**TN**	20	0.513	0.0373	0.599	0.1174	2.429	0.0770
**RC**	7	0.143	0.0020	-	-	-	-
**CV**	18	0.412	0.0011	0.509	0.4145	2.147	0.1683
**TY**	21	0.591	0.0156	0.527	0.2331	1.833	0.3459
**TR**	11	0.401	0.0030	0.488	0.4851	1.816	0.3152
**VN**	17	0.593	0.1290	0.505	0.3014	1.537	0.6252
**LQ**	26	0.675	0.2633	0.500	0.7236	1.444	0.9717
**PPR**	19	0.884	0.6979	0.329	0.8861	1.450	0.5894
**QD**	14	0.778	0.2225	0.500	0.5000	1.499	1.4985
**CB**	8	1.129	0.8322	-	-	-	-
**SJ**	11	0.526	0.1135	-	-	-	-
**LP**	18	0.684	0.2467	0.596	0.1556	2.499	0.1072
**mean**	16	0.611	0.2137	0.506	0.4241	1.850	0.5220
Dominant *Picea chihuahuana* M. trees							
**TN**	13	0.847	0.4424	0.407	0.7033	0.658	0.8830
**RC**	4	0.776	0.6267	-	-	-	-
**CV**	4	1.161	0.8712	-	-	-	-
**TY**	7	0.892	0.5378	-	-	-	-
**TR**	1	-	-	-	-	-	-
**VN**	10	1.022	0.9182	0.318	0.7115	0.968	0.7165
**LQ**	9	0.426	0.1269	0.385	0.9103	1.594	0.6687
**PPR**	4	0.729	0.5331	-	-	-	-
**QD**	9	1.274	0.9744	-	-	-	-
**CB**	3	-	-	-	-	-	-
**SJ**	1	-	-	-	-	-	-
**LP**	6	0.467	0.0960	-	-	-	-
**mean**	6	0.844	0.5696	0.370	0.7750	1.073	0.7561

Note: Significant results after Bonferroni correction are shown in bold type. *CE*, $\bar{W}$ and $\bar{R}$ failed in some plots because of insufficient numbers of trees (repetitions) for the calculations.

**Table 7:** Analysis of spatial structure of the suppressed and dominant trees in 50 x 50 m plots containing all tree species(species shown [44]),in the 12 study locations, based on the neighbourhood-based Clark-Evans index (*CE*), Uniform Angle index ($\bar{W}$), and Mean Directional index ($\bar{R}$). *P* values estimated with 10,000 permutations. *N* equals the tree number in the plot. 1) La Tinaja (*TN*), 2) El Ranchito (*RC*), 3) El Cuervo (*CV*), 4) Talayote (*TY*), 5) Las Trojas (*TR*), 6) El Venado (*VN*), 7) La Quebrada (*LQ*), 8) Paraje Piedra Rayada (*PPR*), 9) Quebrada de los Durán (Arroyo del Indio Ignacio) (*QD*), 10) Cebollitas (*CB*), 11) San José de las Causas (*SJ*), and 12) La Pista (Arroyo de La Pista) (*LP*).

Location	*N*	*CE*		*1-P*(*Z* ≥ *CE*)	$\bar{W}$	*P*(*Z*≥$\bar{W}$)	$\bar{R}$		*P*(*Z* ≥$\bar{R}$)
**Suppressed trees (from all tree species)**									
**TN**	62	0.701		0.0060	0.498	0.4006	1.963		0.1542
**RC**	76	**0.656** ^**cl**^		**0.0000**	0.546	0.0582	2.018		0.0901
**CV**	70	0.721		0.0047	0.590	0.0033	**2.367** ^**cl**^		**0.0002**
**TY**	46	0.726		0.0196	0.515	0.2634	1.733		0.5456
**TR**	80	**0.696** ^**cl**^		**0.0002**	0.547	0.0819	2.043		0.1076
**VN**	53	0.760		0.1152	0.608	0.0172	2.045		0.3578
**LQ**	100	0.802		0.0482	0.532	0.1127	1.980		0.1077
**PPR**	31	0.788		0.3142	0.506	0.3482	1.746		0.4744
**QD**	106	**0.689** ^**cl**^		**0.0001**	0.536	0.0829	2.104		0.0107
**CB**	75	0.830		0.1421	0.554	0.0617	2.177		0.0215
**SJ**	73	0.897		0.4779	0.475	0.6896	1.763		0.5202
**LP**	80	0.862		0.1763	0.506	0.4149	1.892		0.3110
**mean**	71	0.761		0.1088	0.533	0.2177	1.981		0.2358
**Dominant trees (of all tree species)**									
**TN**	14		0.902	0.4830	0.407	0.6805	0.658	0.9014	
**RC**	11		1.004	0.9572	0.425	0.7566	1.568	0.6909	
**CV**	12		0.795	0.4340	-	-	-	-	
**TY**	12		0.833	0.4154	0.478	0.3358	1.789	0.1892	
**TR**	9		1.099	0.8769	-	-	-	-	
**VN**	12		0.889	0.8473	0.541	0.2768	1.510	0.7192	
**LQ**	12		0.492	0.0981	0.360	0.9360	1.147	0.8622	
**PPR**	7		0.964	0.6680	0.500	0.1914	1.610	0.1149	
**QD**	27		0.867	0.5487	0.449	0.8022	1.653	0.7132	
**CB**	13		0.989	0.7451	-	-	-	-	
**SJ**	17		0.955	0.0449	0.370	0.3697	0.832	0.8318	
**LP**	11		0.708	0.1647	-	-	-	-	
**mean**	13		0.875	0.5236	0.441	0.5436	1.346	0.6279	

Note: Significant results after Bonferroni correction are shown in bold type. ^**cl**^ indicates a clustering pattern in the plot. $\bar{W}$ and $\bar{R}$ failed in some plots because of insufficient numbers of trees (repetitions) for the calculations.

The covariations *C[CE x N]*, *C[* $\bar{W}$ *x N]*, *C[* $\bar{R}$ *x N], C[CE x G]*, *C[* $\bar{W}$ *x G]*, *C[* $\bar{R}$ *x G], C[fcd x CE]*, *C[fcd x* $\bar{W}$ *]*, *C[fcd x* $\bar{R}$ *], C[CE x υ*_*sp*_ *]*, *C[* $\bar{W}$ *x υ*_*sp*_ *]*, and *C[* $\bar{R}$ *x υ*_*sp*_ *]* were not statistically significant. The strongest covariation (*C*) between spatial pattern indices and tree density was *C[* $\bar{R}$ *x N]* with + 0.61 (*P* = 0.07) (i.e. clustering tended to be associated, but not significantly, with high stand density). The strongest covariation (*C*) between spatial pattern indices and tree species diversity was *C[* $\bar{W}$ *x υ*_sp,2_ *]* with + 0.51 (*P* = 0.12) (i.e. clustering tended to be associated, but not significantly, with high tree species diversity). None of the 12 uneven-aged forest plots showed a statistically significant regular spatial tree pattern.

No statistically significant differences (*Diff*) (*P* < 0.01) between the average spatial pattern indices (*CE*, $\bar{W}$ and $\bar{R}$) were observed for i) *P*. *chihuahuana* and all tree species or ii) suppressed and dominant *P*. *chihuahuana* trees.

When latitude was analyzed, we found that after Bonferroni correction, *CE*, but not $\bar{W}$ and $\bar{R}$, decreased significantly from the southern to northern plots containing all tree species (C = -0.97, *P* = 0.0008).

## Discussion and Conclusions

In this study, we analysed the fine-scale spatial tree patterns in a special forest tree community of *P*. *chihuahuana* M. in Mexico. We examined the spatial tree pattern and its relationships to tree stand density, vertical structure, tree species diversity, geographical latitude and tree dominance.

The findings demonstrate that the hypothesis of aggregated tree pattern is not applicable to the mean pattern measured by *CE*, $\bar{W}$ and $\bar{R}$ of the uneven-aged *P*. *chihuahuana* trees and *P*. *chihuahuana* tree community (Tables 3 and 5) and but to specific spatial scales measured by the univariate *L*-function, because 58% of the plots showed clustering at small (42%), intermediate and larger scales (17%) (Table 4). Frequent clustering at small scales was mainly caused by a high degree of aggregation of trees smaller than 23.2 cm DBH (Table 4) [42]. We also found that the mean $\bar{W}$ and $\bar{R}$ values for immature (suppressed) were significantly larger than the corresponding values for mature (dominant) trees (Table 7). We also observed that immature *P*. *chihuahuana* trees showed clustering in 25% of the plots, according to the *CE* index. As in many primeval forests, small (young) individuals are almost always located in groups (Table 4)—often as a result of tree fall gaps in the canopy as the dominant type of disturbance [61][62]. The common clustering at small scales in the *P*. *chihuahuana* tree community indicates that the forest patches were often created only by one canopy tree falling, as typically observed in species-rich tropical rain forests [63] and occasionally in old-growth (subalpine) spruce-fir forests. These forests tend to be horizontally structured, mainly because an initiating disturbance is followed by long periods when small-scale, low-intensity disturbances control tree regeneration [8]. Therefore, fire disturbance, which almost always homogenizes stands, is a rare event in the *P*. *chihuahuana* tree community and should not be necessary for or beneficial to the community dynamics. Moreover, fires may bring an end to this fragmented tree community in very small and isolated locations [1] [17]. In contrast, the clustering by small-scale disturbances may be mainly caused by insect attack, disease or windthrow, which may create patchiness and spatial heterogeneity within locations [8].

The rare clustering at larger scales was mainly affected by the low tendency of aggregation of canopy trees (Table 4), as also reported by Malik et al. [64], Christensen [65] and Whipple [66] for uneven-aged populations. Therefore, the overall random patterns were a result of shift from initial aggregation to a random distribution [67]). Hence, Lepš and Kindlmann [42] postulated that i) an observed random pattern does not represent evidence of independence of individuals and ii) the intensity of spatial pattern should not be considered a measure of community organization.

Self-thinning due to intra and inter-specific competition-induced mortality was probably the main cause of the decrease in aggregation intensity [42], [67] during the course of population development in the *P*. *chihuahuana* tree community. However, environmental heterogeneity, uneven-age distributions, insufficient competition, limited seed dispersal and random germination may have prevented the presence of a significantly regular pattern of the mature trees in the tree community under study [68]. The aforementioned factors, particularly insufficient competition in the plot may also have favoured clustering in the northern locations (plots).

The number of *P*. *chihuahuana* trees in the neighbourhood of other tree species (or the number of trees from other species in the neighbourhood of *P*. *chihuahuana*) was not expected. The spatial distribution of *P*. *chihuahuana* trees was independent of the patches of other tree species, except in the San Jose de las Causas (SJ) location (Table 4, Fig 3). In SJ, spruces had a repulsive pattern to other species, similarly to a study in an old growth spruce-fir forest in Changbaishan Natural Reserve, China [41]. We therefore assume that there was a similar but weaker inter- and intraspecific competition between the trees at the species level [68] and that *P*. *chihuahuana* can tolerate partial shade conditions. The bivariate *L*-function showed that smaller trees (of all species) often grew in the neighbourhood of larger trees (of all species) (Table 4), typically in uneven-aged forests (Fig 2) and probably as a result of some shade-tolerant frequent tree species under mature canopy (such as *Abies durangensis*, *Cupressus lindleyi* and *Juniperus deppeana*) and the slender shaped crowns of the mature canopy trees in this community [69]. The smaller *P*. *chihuahuana* trees, did not, however, generally grow more frequently in the surroundings of larger *Picea* trees (Table 4), perhaps only for statistical reasons (such as few tree/repetitions) or due to no special preferences of the young (small) *Picea* trees for mature canopy *Picea* trees. In this study, *P*. *chihuahuana* regeneration was occasionally found on horizontal dead trees.

The plots in which a clustered structure was observed tended to be associated (not significantly) with a large number of trees because of the presence of a greater number of understory trees. Understory trees often displayed a tendency to grouping (Table 4).

Although the aggregation indices were not associated with the diameter distribution, the results showed that none of the 12 uneven-aged forest plots under study displayed a statistically significant regular spatial tree pattern; however, 58% showed clustering in specific spatial scales at the 0.1% significant level (Table 4). No covariation (*C*) between aggregation indices and diameter distributions was observed, because the 12 diameter distributions scarcely varied in their reverse *J*-shaped form (Table 2)

The cluster structure was weakly positively related to higher tree species diversity, probably due to a combination of the accumulation effect [13] [70] and increasing competition in denser plots [71]. While the accumulation effect resulted in higher diversity, the self-thinning processes led to saturation in tree species diversity [72].The high tree species diversity in the *P*. *chihuahuana* community [13] may also provoke clustering at smaller scales (in small gaps) because the lifespan and dimension of each tree species are often different. The probability that two or more trees of different species would fall at the same time and create a gap is lower than the probability of the same happening with trees of the same species.

We conclude that satisfactory understanding of spatial forest structure is essential for the sustainable conservation of this unique mixed uneven-aged *Picea* forest [20]. Our measures of spatial tree structure, particularly $\bar{W}$ and $\bar{R}$ failed in several plots because of an insufficient number of trees (repetitions) for the calculations. Therefore, we recommend use of larger sample plot sizes (> 0.25 ha) in uneven-aged and species-rich forest ecosystems to detect less obvious, but important, relationships between spatial tree pattern and functioning and diversity in these forests.

## Supporting Information

S1 Dataset Data set used in this study. (XLS)
