# Correction: Spatial Distribution Patterns in the Very Rare and Species-Rich *Picea chihuahuana* Tree Community (Mexico)

**DOI:** 10.1371/journal.pone.0143899

**Published:** 2015-11-23

**Authors:** Christian Wehenkel, João Marcelo Brazão-Protázio, Artemio Carrillo-Parra, José Hugo Martínez-Guerrero, Felipe Crecente-Campo

Fig 1 is incorrect. The authors have provided a corrected version here.

**Fig 1:**
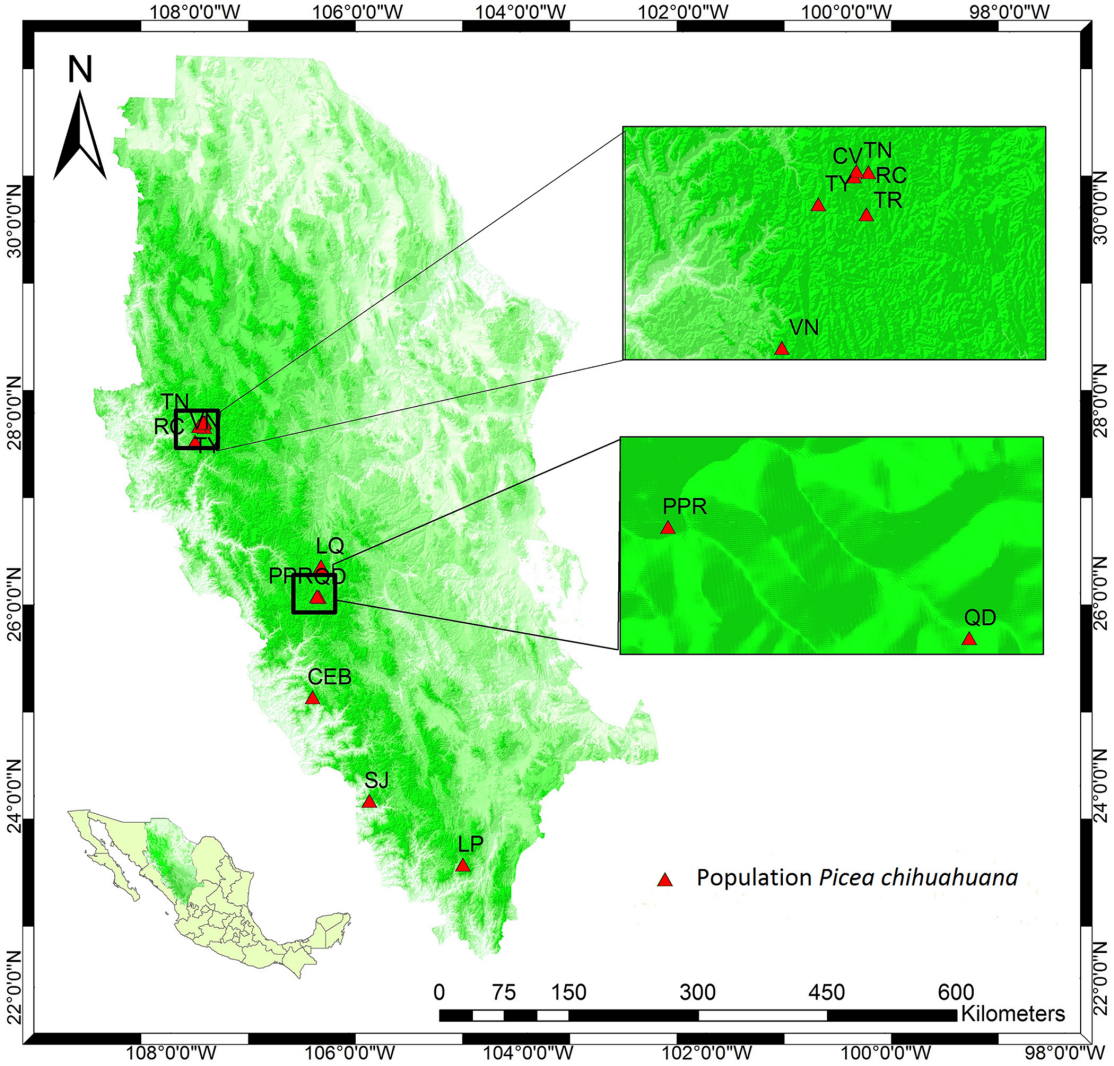
Location of the study area on the Sierra Madre Occidental, Durango (Mexico). Map of the 12 locations of the *Picea chihuahuana* Martínez tree community under study in the States of Durango and Chihuahua (Mexico): 1) La Tinaja (TN), 2) El Ranchito (RC), 3) El Cuervo (CV), 4) Talayote (TY), 5) Las Trojas (TR), 6) El Venado (VN), 7) La Quebrada (LQ), 8) Paraje Piedra Rayada (PPR), 9) Quebrada de los Durán (Arroyo del Indio Ignacio) (QD), 10) Cebollitas (CB), 11) San José de las Causas (SJ), and 12) La Pista (Arroyo de La Pista) (LP).Data sources: Own compilation based on freely-accessible digital maps from INEGI, Mexico (http://www.inegi.org.mx/geo/contenidos/mapadigital/).

In the Material and Methods section, there is an error in the sixth equation of the section titled “Covariation analysis.” Please view the complete, correct equation here:

$$C:=\frac{\sum_{i<j}(X_{i}-X_{j})\cdot (Y_{i}-Y_{j})}{\sum_{i<j}|(X_{i}-X_{j})\cdot (Y_{i}-Y_{j})|}$$

There is an error in the third sentence of the fifth paragraph in the Discussion and Conclusions. The correct sentence is: In SJ, spruces had an aggregated pattern to other species, contrarily to a study in an old growth spruce-fir forest in Changbaishan Natural Reserve, China [41].
